# The influence of storage temperature on fracture behavior of cryopreserved teeth—An in vitro study

**DOI:** 10.1002/cre2.283

**Published:** 2020-03-28

**Authors:** Johannes Angermair, Dirk Nolte, Robert Linsenmann, Karl‐Heinz Kunzelmann

**Affiliations:** ^1^ Clinic of Oral‐ and Maxillofacial Surgery, Translational Implantology Medical Center Freiburg – Faculty of Medicine, University of Freiburg Freiburg Germany; ^2^ Private Practice for Oral and Maxillofacial Surgery Munich Germany; ^3^ Department of Conservative Dentistry and Parodontology University Medical Center Munich Munich Germany

**Keywords:** critical loading, cryopreservation of teeth, fracture, storage temperature

## Abstract

**Objectives:**

Cryopreservation is discussed as a viable method of preserving teeth for determined autogenous tooth transplantation. Unchanged physical properties of hard tooth tissues are crucial for functional healing. Due to different thermal expansion coefficients of enamel and dentin or the crystallization process, the freezing process may lead to crack formation, which could adversely impact the long‐term prognosis of the teeth.

**Material and methods:**

Twenty third molars (*n* = 20) were frozen slowly using a conservative cryopreservation protocol and stored at −80°C (group 1) and −196°C (group 2). After a storage time of 2 weeks, the samples were thawed to a temperature of +36°C and embedded in polymethyl methacrylate blocks. Cyclic loading was carried out using a spherical steel test specimen with 50,000 mechanical load cycles, followed by load to failure testing for determination of critical load.

**Results:**

No significant difference in the first load drop could be detected during the load to failure test under different storage conditions. The values until fracture correlated very closely in contralateral tooth pairs, which emphasizes the importance of crown geometry in load to failure tests.

**Conclusions:**

Conclusions: Cryopreservation, specifically the storage temperature, does not appear to have a significant effect on the physical properties of tooth transplants.

## 1 INTRODUCTION

Autogenous tooth transplantation (autoTX) is valid option of tooth replacement with sufficient survival rates (Andreasen, Paulsen, Yu, Bayer, & Schwartz, 1990). In check‐ups over 17–41 years, success rates of 95% have been reported (Czochrowska, Stenvik, Bjercke, & Zachrisson, 2002). Furthermore, orthodontic treatment following tooth transplantation is possible, thereby supporting the codevelopment of natural jaw growth in childhood (Paulsen, 2001; Tschammler et al., 2015). Healthy teeth which have to be removed for orthodontic or surgical reasons are ideally suited as donor teeth for autoTX (Plakwicz, Wojtowicz, & Czochrowska, 2013; Zachrisson, 2004). Since there is often no need for the extracted teeth at the time of extraction, cryopreservation could allow them to be transplanted for a subsequent trauma or tooth loss (Osathanon, 2010; Schwartz, 1986). During this process, the tooth transplants are frozen at −196°C under controlled conditions. This slows down the chemical processes so that the biological time is effectively stopped, and the transplants can be stored over several years (Oh et al., 2005).

Maintaining the periodontal ligament (PDL) is essential for the physiological healing of tooth transplants. While *slowly* frozen teeth (0.3–1°C/min followed by plunging in liquid nitrogen) show periodontal regeneration comparable with directly transplanted teeth, Kawasaki et al. identified progressive root resorption on rapidly frozen transplants in animal studies (Kawasaki, Hamamoto, Nakajima, Irie, & Ozawa, 2004; Schwartz, Andreasen, & Greve, 1985). The problem here is attributed to the so‐called ice injury of cells, which occurs as a result of the changing osmotic pressure when ice crystals are formed (Schwartz & Andreasen, 1983). This ice injury can be minimized by slow and controlled freezing processes (Kawasaki et al., 2004; Mazur, 1984; Schwartz et al., 1985). Furthermore, cryoprotectants such as dimethyl sulfoxide (DMSO) minimize crystal formation (Yoshizawa et al., 2014). As the ideal cryoprofile, Schwartz et al. recommend gradually equilibrating the storage medium to 10% DMSO, which should cause the least amount of damage to the PDL (Schwartz et al., 1985; Schwartz & Andreasen, 1983). However, human case reports replanting cryopreserved teeth reported periodontal regeneration in some cases, while a case series by Yoshizawa et al. demonstrated root resorption in 43% of the teeth (Paulsen, Andreasen, & Schwartz, 2006; Schwartz, 1986; Yoshizawa et al., 2014). The regeneration of the periodontium may be promoted by improving cryopreservation protocols including a slow equilibration of cryoprotectants. Therefore, some authors have suggested a slow cryopreservation procedure in a magnetic field in order to prevent ice formation and reduce the concentration of cryoprotectants (Abedini et al., 2011; Kaku et al., 2010).

Another important aspect for the autoTX of cryopreserved teeth is their structural behavior after storage. One problem of cryopreservation is the lack of diffusion of the cryoprotectant into the pulp tissue of the tooth, which may result in intracellular crystallization in the pulp tissue which will not survive cryopreservation, unlike the PDL cells. Mechanical expansion of the pulp tissue during crystallization may contribute to cracks forming in the hard tooth tissue (Kuhl et al., 2012). Furthermore, enamel and dentin exhibit different thermal expansion coefficients. Literature states that the linear thermal expansion coefficient of dentin is 11 × 10^−6/°C, and the coefficient of enamel is 17 × 10^−6/°C (Xu, 1990). As a result of this difference, it can be assumed that the cooling process might induce stress in the enamel‐dentin interface, which could lead to crack formation. The physiological masticatory load on the teeth can lead to further crack growth, which in the long term can result in cracks forming in the enamel, as well as infections if cracks form in the dentin. The aim of this investigation is, therefore, to clarify what effect the storage temperature has on the structural behavior of cryopreserved teeth. Specifically, it is to be tested what differences in load to failure values can be induced through two treatment conditions: cryopreservation of teeth at −80°C and at −196°C.

## 2 MATERIALS AND METHODS

### 2.1 Sampling

In this study, upper third molars were carefully removed from 10 patients (4 male, 6 female, age 15–24, median: 18.7) through careful distal luxation without using a forceps. Immediately after extraction, all teeth were stored in a suitable growth medium (Dentosafe®, Dentosafe GmbH, Iserlohn, Germany) in order to prevent them from drying out. Two teeth from one patient were always paired up in order to largely exclude interindividual differences with regard to fracture strength, level of fluoridation or tooth shape as sources of error. Contralateral tooth pairs with deviating morphological or anatomical characteristics (number and shape of cusps, height of central fossa) or lesions like caries or enamel fluorosis where excluded from the study. Only impacted teeth were selected in order for crack formation resulting from prior damage due to caries or mastication to be reliably excluded. All samples were examined under a microscope before use to check for enamel defects that may have occurred during extraction. The patients were also selected according to the maturity of their teeth (at least 2/3 root length), which is essential for stably embedding them in polymethyl methacrylate. All patients agreed in writing to the use of their extracted teeth for research purposes. This study was approved by the Ethics Commission of the Ludwig Maximilian University of Munich (UE No. 082–13).

### 2.2 Cryopreservation of the samples

The teeth were prepared for cryopreservation within 2 hr of being extracted and randomly divided up into two test groups. The samples in group 1 (*n* = 10) were stored at −80°C, and group 2 (*n* = 10) at −196°C. All samples were stored according to a standardized (Schwartz et al., 1985) procedure which we modified. The samples were stored in the growth medium and then in a specially developed freezing medium. After the samples were cooled to +4°C, DMSO (Sigma, St. Louis, MO) was gradually added to the freezing medium at 10–15 min intervals until the recommended concentration of 10% DMSO was achieved. The samples were then cooled from room temperature of 21 to −7°C in a slow freezing process. After completing the same freezing protocol, the two groups were separated. Samples of group 1 were frozen to −80°C in an ultra‐low temperature freezer (U101 Innova^R^, New Brunswick Scientific, Eppendorf AG, Hamburg, Germany), while samples of group 2 were stored at −196°C in the gas phase of liquid nitrogen. All samples were stored under these conditions for 2 weeks. After the 2 weeks were up, all samples were thawed in falcons in a water bath heated to a constant temperature of +36°C at least 6 hr, as described and recommended in literature (Schwartz et al., 1985). Again, microscopic examination for crack formation was performed and no crack development could be assessed after the thawing process.

### 2.3 Embedding

After the teeth were removed from the Falcons, they were embedded with the root in polymethyl methacrylate (Paladon^R^ 65, Heraeus Kulzer, Hanau, Germany) at an even height of 2 mm up to the cementoenamel junction (according to the biological width). The polymethyl methacrylate blocks were of a standardized dimension of approx. 20 mm × 20 mm × 15 mm (*W* × *D* × *H*). The samples were stored in the freezing medium, to prevent them from drying out with possible effects on crack formation. During chewing simulator testing the teeth were stored in saline solution.

### 2.4 Fatigue simulation

**Figure 1:**
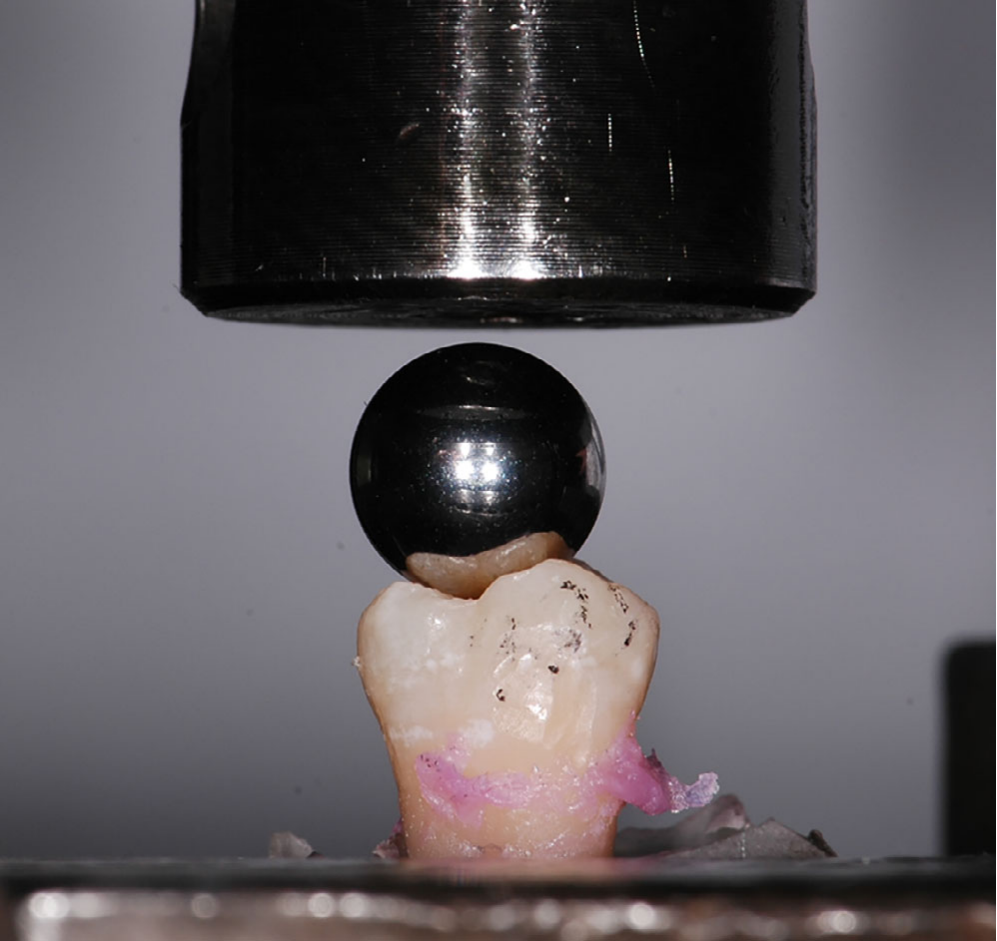
Vertical load‐to‐failure testing with spherical steel specimen. A maximum vertical load was implied until failure occurred

The samples were subjected to a cyclic, vertical load in the chewing simulator to induce crack development according to the crack formation theory (Chai, 2014; Chai, Lee, & Lawn, 2010; Gao, An, Yahyazadehfar, Zhang, & Arola, 2016). The teeth were subjected to a total of 50,000 load cycles, each with a force of 50 N (1 Hz). A 10 mm steel ball was used to apply a vertical, three‐point load to the central fissure on the occlusal surface to simulate the distribution of force according to the mortar‐pestle principle (Figure 1). While the load was being exerted on the teeth, they were stored in saline solution and kept moist during all transfers to avoid drying out.

### 2.5 Load to failure

**Figure 2:**
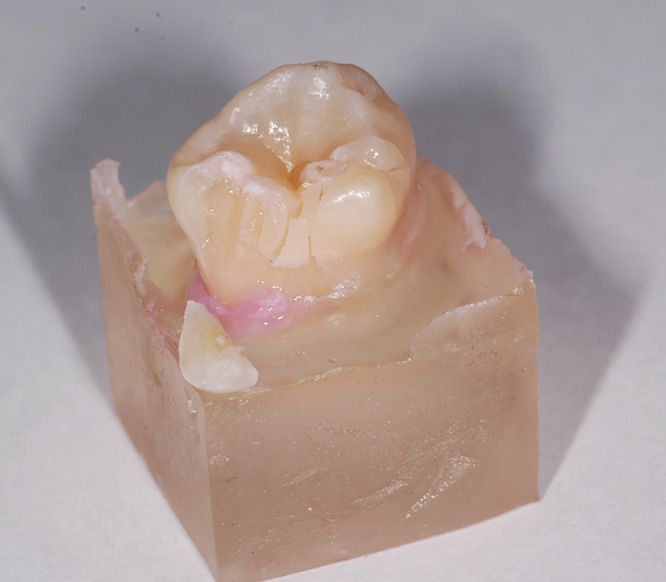
Fractured tooth with chipping of the enamel wall along the dentin core (1)

**Figure 3:**
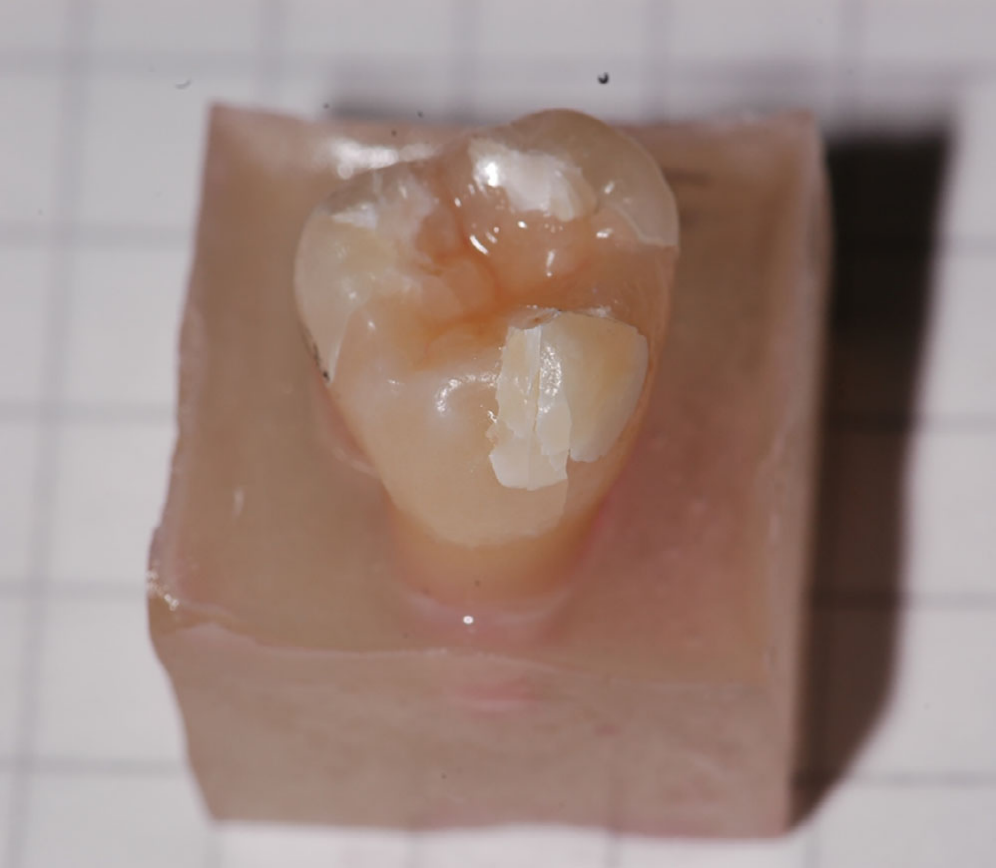
Fractured tooth with crack enamel fracture pattern (2). A distinction is made between fracture patterns where a larger tooth fragment, including dentin or along the enamel‐dentin junction, has developed (1) and fracture line that has only developed in the enamel (2)

**Figure 4:**
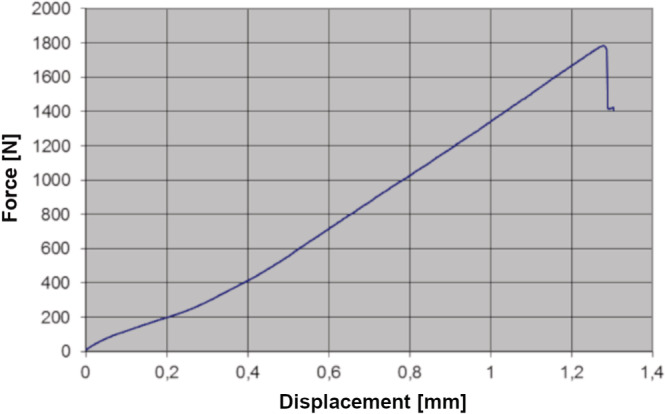
Representative force–displacement curve for tooth 325. After material deformation tooth 325 failed by “Fracture pattern 1.” The peak load before specimen failure (1781.29 N) was documented and taken for further analysis

After cyclic loading in the chewing simulator, the teeth were secured in a universal testing machine (Zwick, Ulm, Germany, type: 1445, crosshead speed: 0.5 mm/min). Using the same spherical test specimen, a vertical three‐point load was applied to the occlusal surface until the teeth fracture (Figures 2 and 3). The maximum load before the load drop was documented in the load curve and used as a basis for further analysis. Since the load–displacement curve of one tooth sample could not be analyzed, there was one less pair of teeth available for the analysis. After load to failure testing, two different fracture patterns were distinguished for further differentiation: “Fracture mode 1” included teeth where a displacement of a large tooth fragment, including dentin or along the enamel‐dentin junction, had developed. “Fracture mode 2” described a fracture pattern in the enamel. Figure 4 displays a representative force–displacement curve for tooth 365 that failed by “Fracture mode 1.”

### 2.6 Statistical analysis

Statistical analysis was performed using the R statistical software. Intraindividual differences were tested with the nonparametric Wilcoxon rank sum test and Shapiro–Wilk test. Different failure modes in the two storage conditions were investigated using the McNemar test. The significance level was set at *p* ≤ .05.

## 3 RESULTS

Table 1 shows a summary of the results per single tooth. In the “Pair” column, the contralateral teeth of the patients were identified with sequential numbers. Each pair of numbers is from the same patient. The “Temp” (temperature) column identifies the storage condition and distinguishes between group 1 and group 2. The maximum fracture load as well as the maximum deformation were gathered from the raw data. In the fracture pattern column, the fracture was divided into a group based on macro photographs.

**Table 1:** Summary of measurement results for each tooth

Tooth number	Pair	Temp. (°C)	Max. load (N)	Max. deformation (mm)	Fracture mode	Stiffness (N/mm)
321	1	−80	488.98	0.13	2	3,729
322	1	−196	662.45	0.23	1	3,036
325	2	−196	1,781.29	1.28	1	1,567
326	2	−80	1,615.51	1.67	1	1,140
327		−80	898.52	0.26	1	3,526
329	3	−196	1,008.55	0.57	1	1958
330	3	−80	1,318.10	0.83	1	1884
331	4	−80	160.67	0.07	2	2,339
332	4	−196	1,389.14	0.44	1	3,833
333	5	−196	800.54	0.17	1	4,927
334	5	−80	542.48	0.23	1	2,250
335	6	−80	840.93	0.20	1	4,608
336	6	−196	645.69	0.23	1	2,975
339	7	−80	333.67	0.10	2	3,660
340	7	−196	581.85	0.18	1	3,557
343	8	−196	1,670.63	0.81	1	2042
344	8	−80	394.19	0.12	2	3,252
346	9	−196	380.47	0.14	2	2,955
347	9	−80	217.78	0.08	2	3,168

*Note*: Descriptive data for all teeth. Contralateral teeth were designated as pairs. Fracture modes “1” and “2” distinguish morphology of fracture lines. The force–deformation curve of tooth number 328 could not be analyzed, therefore there was one tooth less available in the “−196°C" group.

**Figure 5:**
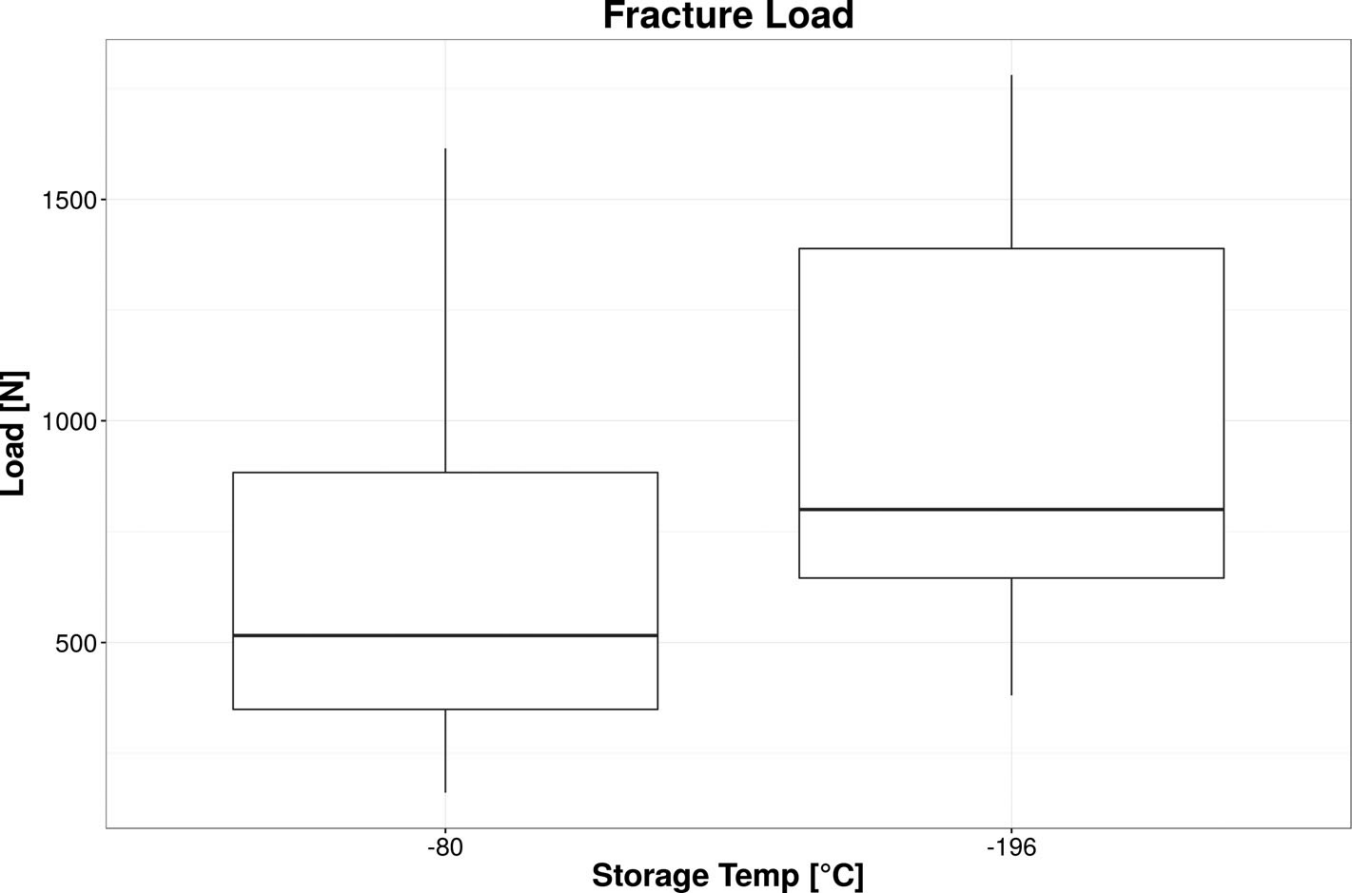
Maximum load‐to‐failure for both storage temperatures. Although mean values (−80°C: 681 ± 483 N; −196°C: 991 ± 506 N) differ, no significant difference could be identified using the Wilcoxon rank sum test (*p* = .094)

The descriptive data are shown in Figure 5 as a box plot. The Shapiro–Wilk test confirms that the maximum fracture loads are normally distributed (*W* = 0.919, *p* = .107). The mean value of the group stored at −80°C is 681 ± 483 N, the mean value of the group stored at −196°C is 991 ± 506 N. The median value of the group stored at −80°C is 516 N, range 161–1,616 N, median of the group stored at −196°C is 801 N, range 380–1,781 N. No significant difference could be identified using the Wilcoxon rank sum test (*p* = .094).

**Figure 6:**
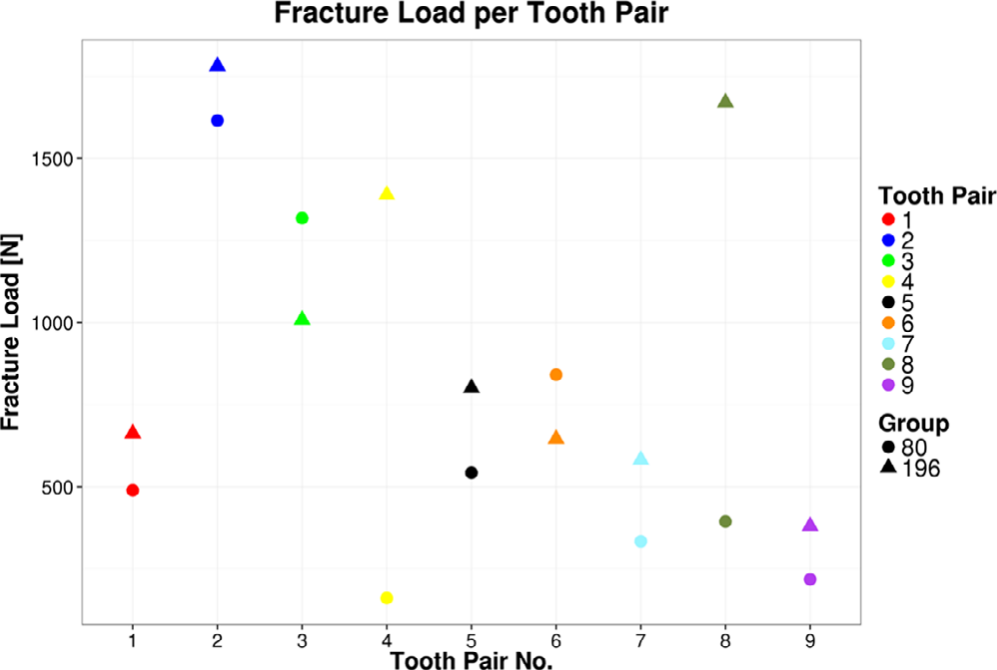
Fracture load (N) for corresponding tooth pairs. Except for pair 4 and 8 the fracture loads and maximum deformation are similar for the contralateral tooth pairs, underlining the importance of intraindividual testing and correlation of crown geometry and maximum fracture loads

**Figure 7:**
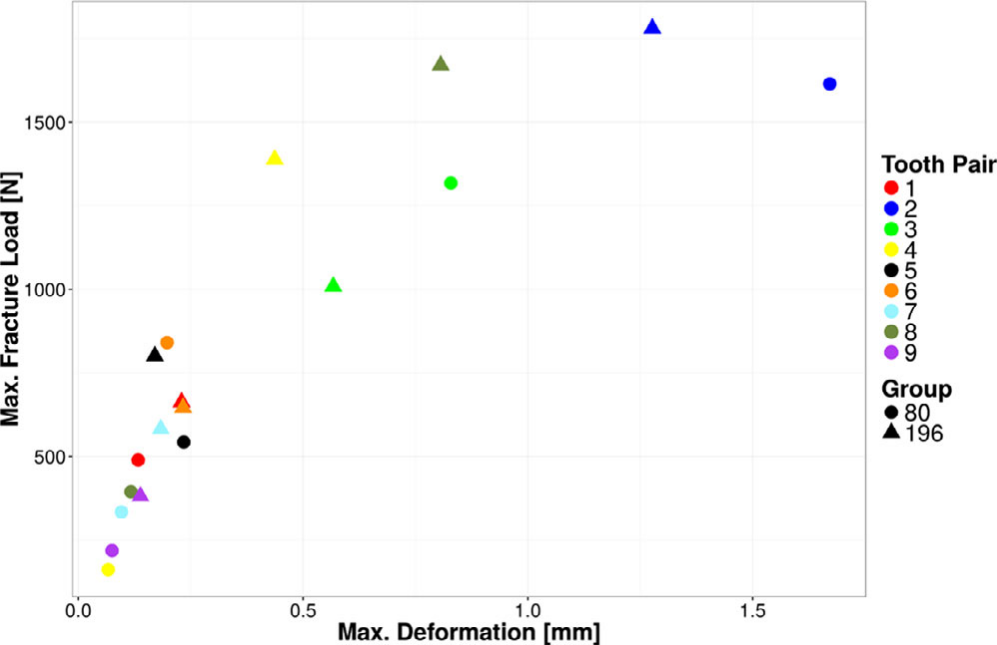
Maximum fracture load as a function of maximum deformation for corresponding tooth pairs

To compare the fracture behavior of contralateral teeth, fracture load and maximum deformation for the individual pairs of teeth were depicted as graphs (Figures 6 and 7). It can be clearly seen in the graphs that the individual pairs of teeth yielded very similar results, and that the interindividual distribution between the different teeth is much larger than within one pair of teeth. The measured values only deviate significantly for the pairs of teeth marked 4 (yellow) and 8 (olive) if the maximum fracture load is taken into consideration. A significant deviation in the energy absorbed before fracture can be identified for pair 8. In terms of the energy absorbed before fracture, pairs 1, 5, 6, and 9 have almost identical values.

Thus, low values of load to failure are concentrated in the samples that failed via “Fracture mode 2,” with a mean load to failure of 1,071 ± 459 N in “Facture mode 1” and 329 ± 121 N in mode 2 (*p* = .001). This observation may help to explain the large difference in load to failure for subjects 4 and 8, as they were characterized by different failure modes in the two storage conditions. “Fracture mode 2” thus was more prevalent in the samples stored at −80°C (*n* = 5) than −196°C (*n* = 1), although this difference is not significant (McNemar test, *p* = .13).

## 4 DISCUSSION

Cryopreservation of teeth allows for a determined autogenous tooth transplantation. Maintaining the vitality of the periodontal cells and the integrity of the hard tooth tissues are crucial for the functional healing of the transplants. While earlier studies assume PDL cell survival through conservative freezing methods (Oh et al., 2005), intracellular crystallization of the pulp tissue may occur due to insufficient diffusion of the cryoprotectant DMSO (Price & Cserepfalvi, 1972). The crystallization could lead to irreversible crack formation in dentin structure. While infraction of the enamel only affects the coronal part of the transplant, cracks in the dentin can reach the root, which may lead to an infection of the root due to direct contact to the periodontal gap, which would affect the long‐term prognosis of the transplants (Kuhl et al., 2012). Furthermore published values for thermal expansion coefficient (dentin: 11 × 10^−6/°C, enamel: 17 × 10^−6/°C) suggest dental hard structures should experience mechanical strain during the cooling or thawing process (Xu, 1990).

Using the standardized method to fatigue the teeth in the chewing simulator and then exert a load until the teeth fracture, no significant differences could be identified between the two storage temperatures. This might be due to the comparatively small difference in thermal expansion between the two materials. Assuming that this difference is temperature independent, a temperature difference of about 220 K (from room temperature to liquid nitrogen boiling temperature) would result in a thermal strain difference of only 0.14%, which is a relatively low strain even for brittle materials such as enamel. As dentin is more compliant than enamel, one might expect the resulting mechanical strain difference to have a more effect on the enamel. Another factor discussed was the effect of crystallization on the crack formation. Since enamel comprising 96% minerals and 4% water and organic materials, the special effect of water freezing can be assumed to be quite low. Thermal strain in it would be proportional to the integral of the thermal expansion coefficient over temperature. If cooling to cryogenic temperatures would cause structural damage due to crystallization tissues with higher water proportion such as dentin and the pulp would be affected.

To identify fracture growth in dentin and enamel a difference was made between a fracture line in the enamel (Fracture mode 2) and the enamel lamellae breaking off with exposure of the dentin (Fracture mode 1). An important finding is that samples that failed via mode 2 showed a lower mean load to failure (329 ± 121 N) than and teeth that failed via mode 1 (1,071 ± 459 N) in mode 1 (*p* = .001). As storage temperature does not significantly affect these values one might assume a higher impact of the facture mode than the storage temperature on critical loading values. In load to failure testing, where an occlusal load is applied to the molars, it can be assumed that overloading will cause the fracture of the enamel wall or enamel, taking into account the common crack formation theories in the enamel (Popowics, Rensberger, & Herring, 2004). The hard, glass‐like enamel sheath absorbs energy, and the flexibility of the softer dentin prevents the fracture from developing into the core. During the trial setup with the load being removed at the first load drop, it is likely that a macroscopic fracture line in the dentin is not created. Consequently, to investigate fractures in the dentin core, microscopic investigations have to be carried out.

These findings are in line with previous studies which could not identify crack growth due to cryopreservation using different approaches. An investigation of the hard tooth tissue using synchrotron radiation‐based microcomputed tomography (SRμCT) carried out by Kuehl et al. showed no cracks in the dentin after cryopreservation at −196°C, but a small number of vertical cracks in the enamel (Kuhl et al., 2012). Due to the crack lines and the position of the cracks in the enamel, the investigators concluded that they were caused by the use of forceps during extraction, rather than stress during cryopreservation. However, under test conditions, only fractures of up to 1.84 μm in size could be identified (Kuhl et al., 2012). Oh et al. also investigated the changes in the physical properties of cryopreserved teeth after being stored for 1 week at −196°C (Oh et al., 2005). Here, the Vickers hardness test was carried out. They could not detect any significant differences in the hardness of the cryopreserved teeth compared to the control group. However, longitudinal fractures could be identified in 25% of teeth after cryopreservation and subsequent loading. After storing the teeth in a wet chamber at +37°C for 1 week, the measured hardness increased in 7 out of 8 control teeth and 6 out of 8 frozen teeth; a significant difference could not be determined. The authors concluded that the hardness of a transplant could improve in the oral environment (Oh et al., 2005). This assumption supports the theory of tufts being stabilized by the penetrating liquid under masticatory function, which could prevent or at least slow down fracture growth (Chai et al., 2010). With Oh et al. choosing a storage time of 1 week and our study testing the teeth after 2 weeks, rather short storage times where chosen. Taking into account the proposed hypothesis of crack development due to thermal expansion during cooling and thawing, this aspect seems to be negligible. However, the effect of storage time on dental tissues could be investigated in further studies.

One main result of the study was that contralateral teeth of one patient showed similar results in the load capacity of the hard tooth substances, while the tooth pairs of different patients show large interindividual distribution of values. Therefore, it can be assumed that intraindividual differences are lower than interindividual differences, which can be attributed to tooth morphology. Geometric properties such as the cusp radius and the thickness of the enamel on the loaded cusp are stated in literature as important variables to determine the maximum load until failure (Lawn & Lee, 2009). Other authors emphasize the prevailing importance of the tooth radius and the cusp radius compared to the enamel thickness (Barani et al., 2011). Similar tooth morphology, which is the case with contralateral teeth, requires a comparable load until complete fracture. It should, therefore, be emphasized that these data also prove how important it is when taking future measurements to use contralateral tooth pairs for comparing two groups. However, morphological variations are described to be high in third molars, which could explain the large interindividual distribution observed (Juhl, 1983).

A particular weakness of the study is the absence of a comparison with noncryopreserved teeth which would have provided a control group. Nevertheless, load to failure values assessed in experimental studies in vitro are comparable. Paulsen et al. described critical loads of 500–850 N for the first load drop with a delamination of the enamel from the dentin by load application with a flat rod at the most prominent cusp of the teeth (Lee et al., 2009). Popovic et al. reported load to failure values for human third molars of 611–1807 N (Popowics, Rensberger, & Herring, 2001) that were tested with a steel ball on the grounded lingual cusps. It seems applicable that tooth geometry and different experimental setup used by other authors have crucial effects on measured fracture strengths. Nevertheless, reported values seem to be in line with the measured values in this study concluding no effect of cryopreservation on the mechanical behavior of teeth.

Physiological loads that are incurred during chewing food (70–150 N) do not reach critical loads assessed in this study (Scully, 2002). For the evaluation of masticatory function of cryopreserved teeth maximal biting forces need to be taken into consideration. Since maximal biting forces of up to 500 N are possible, differing by gender and age (Takaki, Vieira, & Bommarito, 2014; Tripathi et al., 2014), load to failure values in our studies of 516 N (median values) for teeth stored at −80°C, and 801 N for teeth which were stored in liquid nitrogen, show acceptable values. However, in extreme situations, such as patients who suffer from bruxism, values of up to 800 N are reported (Rees, 2006), which appears critical. As experimental setup cannot reproduce elasticity of the parodontal ligament and other factors reducing critical load in real life inserting the tested transplants as a functional prosthesis with normal chewing function can be considered (Barani et al., 2011).

Although the study implies no effect of storage temperature on the physical properties of dental hard tissues, the success of cryotransplants is strongly determined by the survival of PDL. Further study designs to determine ideal cryopreservation protocols should therefore aim for effective preservation of all dental structures.

## 5 CONCLUSIONS

The storage temperature of cryopreserved teeth seems to have no effect on fracture behavior. This may also be due to the modern and conservative cryoprotocols, which allow maximum preservation of the hard tooth tissues. Another important finding is that intraindividual differences were very low while the interindividual differences varied greatly. The maximum load of the teeth until the enamel wall fractures correlates very closely with the values of each contralateral tooth. The importance of crown morphology in relation to the load limit can therefore be concluded. From a physical perspective, cryopreservation can be used safely to store tooth transplants.

## CONFLICT OF INTEREST

The authors report no conflicts of interest.
